# Optimizing exosomal RNA isolation for RNA-Seq analyses of archival sera specimens

**DOI:** 10.1371/journal.pone.0196913

**Published:** 2018-05-08

**Authors:** Emily N. Prendergast, Marcos Abraão de Souza Fonseca, Felipe Segato Dezem, Jenny Lester, Beth Y. Karlan, Houtan Noushmehr, Xianzhi Lin, Kate Lawrenson

**Affiliations:** 1 Women's Cancer Program at the Samuel Oschin Comprehensive Cancer Institute, Cedars-Sinai Medical Center, Los Angeles, California, United States of America; 2 Department of Genetics, University of Sao Paulo, Ribeirao Preto, SP, Brazil; 3 Department of Neurosurgery, Henry Ford Health System, Detroit, Michigan, United States of America; 4 Center for Bioinformatics and Functional Genomics, Samuel Oschin Comprehensive Cancer Institute, Cedars-Sinai Medical Center, Los Angeles, California, United States of America; Institute of Animal Sciences, GERMANY

## Abstract

Exosomes are endosome-derived membrane vesicles that contain proteins, lipids, and nucleic acids. The exosomal transcriptome mediates intercellular communication, and represents an understudied reservoir of novel biomarkers for human diseases. Next-generation sequencing enables complex quantitative characterization of exosomal RNAs from diverse sources. However, detailed protocols describing exosome purification for preparation of exosomal RNA-sequence (RNA-Seq) libraries are lacking. Here we compared methods for isolation of exosomes and extraction of exosomal RNA from human cell-free serum, as well as strategies for attaining equal representation of samples within pooled RNA-Seq libraries. We compared commercial precipitation with ultracentrifugation for exosome purification and confirmed the presence of exosomes *via* both transmission electron microscopy and immunoblotting. Exosomal RNA extraction was compared using four different RNA purification methods. We determined the minimal starting volume of serum required for exosome preparation and showed that high quality exosomal RNA can be isolated from sera stored for over a decade. Finally, RNA-Seq libraries were successfully prepared with exosomal RNAs extracted from human cell-free serum, cataloguing both coding and non-coding exosomal transcripts. This method provides researchers with strategic options to prepare RNA-Seq libraries and compare RNA-Seq data quantitatively from minimal volumes of fresh and archival human cell-free serum for disease biomarker discovery.

## Introduction

Exosomes are extracellular vesicles formed when the plasma membrane fuses with multivesicular bodies produced by inward budding late endosomes [1]. Exosomes are ~30–100 nm in size and are found in many tissues and in a diverse array of biological fluids including serum, plasma, amniotic fluid, ascites, bile, breast milk, cerebrospinal fluid, nasal secretions, saliva, semen, tears, and urine [2–12]. In addition, most cell types secrete exosomes *in vitro* [7, 13].

Exosomes carry proteins, lipids, and nucleic acids [14]. Proteomic studies have isolated thousands of proteins in exosomes [15] including both ubiquitous and cell-type specific proteins that reflect the cell-of-origin [13]. Several members of the tetraspanin protein family, including CD9, CD63, CD81 and CD82, are highly enriched in almost all exosomes isolated from different types of cells *in vitro* and *in vivo* [13,16]. Transmembrane integrins displayed on the surface of exosomes determine the target cell type and contribute to organotropic metastasis in cancer [17]. Lipidomic analyses have identified the predominant species of lipids in exosomes, the function of which are mostly unknown [18]. In addition, recent evidence shows that exosomes contain abundant nucleic acids [15]. Single and double stranded genomic DNA as well as mitochondrial DNA has been described [19–21] and transcriptomic analyses have catalogued both protein-coding messengers and non-coding RNAs (ncRNAs) packaged within exosomes [15]. Exosomal messenger RNAs (mRNAs) are transported *via* exosomes and can be translated into proteins once brought into recipient cells, indicating exosomal mRNAs play a regulatory role in non-cell autonomous signaling [22]. Among the small ncRNAs, microRNAs (miRNAs) are the most abundant and consequently, have been the focus of most exosome biomarker research and functional studies [23, 24]. Other small exosomal ncRNAs include piwi-interacting RNAs (piRNAs), ribosomal RNAs (rRNAs), small nuclear RNAs (snRNAs), and small nucleolar RNAs (snoRNAs) [23–27]. In addition, long ncRNAs (lncRNAs), long intergenic RNAs (lincRNAs) and circular RNAs are also present in exosomes [23, 25, 27, 28].

Stabilized within RNA-protein complexes and shielded by lipid bilayer, exosomal RNAs represent a promising source of diagnostic and prognostic biomarkers for human diseases [29, 30]. Since the identification of RNAs in exosomes in 2007 [22], profiling of exosomal RNAs by RNA-sequencing (RNA-Seq) has been performed on a handful of exosomal preparations from cell lines, and body fluids including plasma, urine and serum [26, 31–33]. However, it is technically challenging to perform enrichment and molecular profiling of exosomes [34], particularly in scenarios where individual clinical samples are limited, and where large numbers of samples need to be processed in a uniform and efficient manner. Here, we describe a detailed protocol encompassing exosomal purification and validation from human cell-free serum, exosomal RNA extraction and preparation of exosomal RNA-Seq libraries from fresh and archived sera specimens.

## Materials and methods

### Preparation of human cell-free serum

For fresh specimens, blood samples were collected from six healthy subjects *via* the antecubital vein. Plasma was immediately isolated by centrifugation at 3,000 g for 10 min at room temperature. Supernatants were collected and centrifuged at 4,000 g for 15 min at room temperature to remove trace number of cells. Cell-free sera were used immediately, or aliquoted and stored at -80°C. Before isolation of exosomes, each aliquot was centrifuged at 8,000 g for 30 mins then 12,000 g for 30 min at 4°C, to ensure complete removal of cell debris and shedding microvesicles. Archival blood samples from 105 patients were collected as part of the Women’s Cancer Program Biobank at Cedars-Sinai Medical Center (CSMC). Only female participants over 18 years of age were included in the study. All underwent surgery at CSMC for a variety of gynecological conditions including but not limited to fibroids, endometriosis and ovarian cancer. All samples were collected with informed patient consent, and specimen collection and exosome characterization was performed with the approval of the CSMC Institutional Review Board.

### Enrichment of exosomes from human sera

#### Isolation of exosomes using ExoQuick

Cell-free serum with a volume of 500 μL was thawed on ice. Serum was mixed with 126 μL of ExoQuick (System Biosciences, catalogue number: EXOQ5A-1). The mixture was incubated at 4°C for 2 h and centrifuged at 1,500 g for 30 min at 4°C. Each specimen was centrifuged twice at 12,000 g for 2 min to remove any additional supernatant. Once isolated, pelleted exosomes were resuspended in 150 μL nuclease-free water.

#### Isolation of exosomes by ultracentrifugation

Cell-free serum with a volume of 500 μL was thawed on ice, overlaid onto a 15% sucrose cushion and ultracentrifuged at 150,000 g for 16 h at 4°C using SW60Ti rotors (Beckman Coulter). Pelleted exosomes were resuspended in 100 μL 1×PBS by overnight incubation at 4°C and then were ultracentrifuged at 150,000 g at 4°C for 2 h. Pelleted exosomes were resuspended in 150 μL of nuclease-free water.

### Transmission electron microscopy (TEM)

Specimens were placed onto freshly glow-discharged 300 mesh carbon formvar grids and set for 10 min. The solution was wicked away with filter paper, and 5 μL 2% glutaraldehyde was placed on the grid for 3 min. The grid was wicked dry, rinsed with water and stained with 2% uranyl acetate for 1 min. Each grid was then wicked and air-dried for 10 min before examining at 80 kV on a JEOL 100CX transmission electron microscope at Electron Microscopy Core Facility of Brain Research Institute at University of California, Los Angeles (UCLA). Images were obtained via a chamberscope film camera.

### Western blotting

To prepare exosomes for western blotting, exosomes isolated by ExoQuick were layered over a 15% sucrose cushion and were ultracentrifuged at 150,000 g at 4°C for 2 h. Pelleted exosomes were resuspended in 150 μL nuclease-free water overnight at 4°C. Exosomes isolated by ultracentrifugation did not require additional processing. Each sample was mixed with 4×Laemmli Sample Buffer and heated at 95°C for 10 min before being loaded for electrophoresis. The membrane was incubated with a primary rabbit anti-CD63 antibody (1:1000 dilution, Santa Cruz Biotechnology, catalogue number: sc-15363, RRID: AB_648179) overnight at 4°C on a shaker. Membranes were washed with TBS/0.2% Tween 20 three times at 15-min interval. The goat anti-rabbit IRDye 680 RD secondary antibody (1:10,000 dilution, Li-COR, catalogue number: 925–68071) was then incubated with the membrane for 1 h at room temperature. Blots were imaged using the Li-COR Odyssey scanner after wash.

### Exosomal RNA extraction

#### Exosomal RNA isolation via TRIzol LS and the RNeasy Mini Kit

Exosomal RNAs were isolated using a combination of phase separation and the RNeasy Mini Kit (Qiagen, catalogue number: 74106). TRIzol LS reagent (500 μL) (Thermo Fisher Scientific, catalogue number: 10296–028) and chloroform (200 μL) were added to each sample. Samples were mixed thoroughly by shaking for over 30 secs and incubated at room temperature for 10 min. Phase separation was performed by centrifugation at 12,000 g at 4°C for 15 min. The upper aqueous phase was collected. Then RLT buffer (3.5×volume), absolute ethanol (2.5×volume), and sodium acetate [3M, pH 5.5] (0.1×volume) (Ambion, catalogue number: AM9740) were added. RNA was extracted using the RNeasy Mini Kit, according to the manufacturer’s instructions. RNA was quantified using the Nanodrop ND-1000 (Thermo Fischer Scientific).

#### Exosomal RNA isolation *via* RNeasy Mini Kit

The RNeasy Mini Kit was used to extract exosomal RNAs according to the manufacturer’s protocol. RNA was quantified using the Nanodrop.

#### Exosomal RNA precipitation

Exosomal RNAs were phase-separated as described above. The upper aqueous phase was collected and 10% volume of sodium acetate (3 M, pH 5.5) was added to each sample. Four microliters glycogen (5 mg/mL, catalogue number: AM9510) and 2.5 times the acquired aqueous volume of absolute ethanol were added. Samples were mixed, incubated overnight at -80°C and then centrifuged at 16,000 g at 4°C for 30 min to pellet RNA. The pellet was washed with 70% ethanol and centrifuged at 16,000 g at 4°C for 5 min. Ethanol was aspirated and the pellet was centrifuged again to remove additional supernatant. Pellets were dried for 5 min before being resuspended in 32 μL of nuclease-free water and purified RNA was quantified using Nanodrop.

#### Exosomal RNA isolation via AllPrep DNA/RNA Mini Kit

The AllPrep DNA/RNA Mini kit (Qiagen, catalogue number: 80204) was used to extract exosomal RNAs following the manufacturer’s protocol. RNA was quantified using the Nanodrop.

### RNA-Seq library preparation and next-generation sequencing

Twenty nanograms exosomal RNA was used to prepare each RNA-Seq library. External RNA Controls Consortium (ERCC) spike-ins (Thermo Fischer Scientific, catalogue number: 4456740) were added as control for normalization of the samples. Strand-specific RNA-Seq libraries were constructed using the NEBNext Ultra Directional RNA Library Prep Kit (NEB, catalogue number: E7420) with the following adjustments to the manufacturer’s instructions: fragmentation was performed for 1 min at 94°C given the size range of our exosomal RNA was typically smaller than 1000 bp (data not shown), and PCR was set to 15 cycles. A unique index was ligated to each sample using the NEBNext Multiplex Oligos for Illumina Index Primers Sets 1–4 (NEB, catalogue numbers: E7335S, E7500S, E7710S, and E7730S). The resulting library concentrations were quantified using the Nanodrop, a Library Quantification Kit (Kapa Biosystems, catalogue number: KK4824), and the Bioanalyzer 2100 (Agilent). The KAPA Kit contains Library Quantification DNA Standards 1–6 (a 10-fold dilution series of a linear, 452 bp template) and a Library Quantification Primer Premix. The sequences of primers are 5'-AATGATACGGCGACCACCGA-3' and 5'-CAAGCAGAAGACGGCATACGA-3'. Libraries were sequenced to generate single-end 75 bp reads on NextSeq 500 platform (Illumina) in high output running mode. Sequencing was performed at the Molecular Genomics Core facility at the University of Southern California.

### RNA-Seq data processing, alignment and analysis

Raw data were downloaded as FASTQ files and QC performed using MultiQC [35]. Around 10 million reads were generated for each sample. The raw data was pre-processed using Trimgalore version 0.4.2 [36] to remove Illumina adapters and reads shorter than 20 bp. Trimmed reads were aligned to version hg38 of the human genome reference sequence using STAR [37]. Mapped reads were then matched to genomic features (i.e. mRNAs and lncRNAs) annotated in GENCODE25 [38] and ERCC annotations using Rsubread package, version 1.24.0 [39], to generate raw counts for each gene. Raw read count data were normalized to ERCC spike-in values across samples using Bioconductor package RUVSeq_1.8.0 [40]. We removed non-expressed genes by requiring more than 5 reads for each gene. R Bioconductor package NOISeq was used evaluate and access the quality control of the count data [41].

### Statistical analysis

Paired Student’s *t* tests were used to calculate *P*-values when comparing methods for exosomal RNA extraction. One-way ANOVA was applied to calculate a *P*-value when contrasting exosomal RNA extraction from different serum volumes. Spearman’s rank correlations were utilized to decide significance of the correlations between RNA yield or OD_260_/OD_280_ with storage time. *P*-values smaller than 0.05 were considered statistically significant.

### RNA-Seq data availability

Exosome RNA-Seq data are publicly available at: https://www.ncbi.nlm.nih.gov/sra/SRP142765.

## Results

### Enrichment of exosomes by ultracentrifugation and ExoQuick

**Fig 1:**
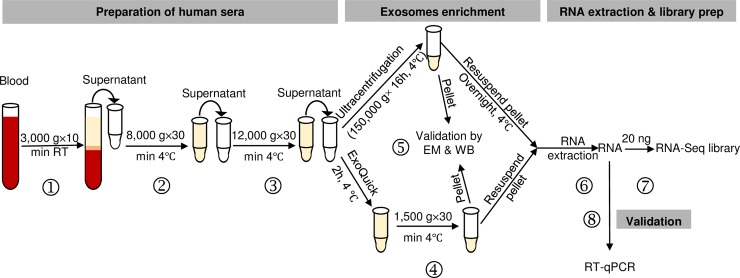
Flow chart for evaluation of exosomal RNAs from cell-free sera as biomarkers for human diseases. Graphic summary of the workflow including time allotment for preparation for cell-free serum (steps ①-③), comparison of methods for exosome enrichment (step ④), validation by transmission electron microscopy (TEM) and immunoblotting for CD63 or other exosomal markers (step ⑤), RNA extraction (step ⑥), and preparation of RNA-Seq libraries (step ⑦). 10–100 nanograms RNA can be used for library preparation with the NEBNext Ultra Directional RNA Library Prep Kit. Step ② is an optional centrifugation step that can be included to ensure the most efficient removal of trace amounts of cell debris and shedding microvesicles. A validation step can be performed with RT-qPCR for specific candidate RNA following RNA-Seq analysis (step ⑧).

**Fig 2:**
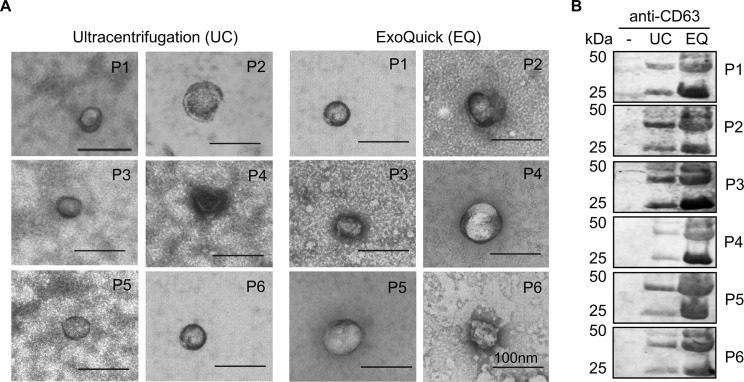
Validation of exosome enrichment from human cell-free sera. (A) TEM micrographs of exosomes in ultracentrifugation (UC) and ExoQuick (EQ) preparations. Data for 6 independent patient samples are shown (P1-6). Exosomes confirmed by size (30-100nm) and appearance. Scale bar in each image represents 100 nm. (B) Immunoblot of CD63 in unprocessed cell-free serum alone (-), UC, and EQ exosomal preparations.

A workflow for exosomal enrichment and validation, RNA extraction, and RNA-Seq library generation is illustrated in Fig 1. Blood samples from six healthy subjects were used to optimize the methods for exosome purification and exosomal RNA extraction. Exosomes were purified from equal volumes of cell-free serum using ultracentrifugation or the ExoQuick reagent and two approaches were then used to validate each approach. First, TEM was used to identify vesicles ranging from 30–100 nm in size. Exosomes were present in all preparations generated using both the ultracentrifugation and ExoQuick protocols (Fig 2A). Second, as CD63 is found on the surface of almost all exosomes, we performed Western blotting for CD63 on all samples. CD63 was detected in exosome preparations from all samples using both protocols, but not unprocessed serum (Fig 2B). Immunoblotting results detected more CD63 in ExoQuick-isolated exosomes for all six subjects, suggesting ExoQuick enriches sera exosomes more efficiently than ultracentrifugation.

### Comparison of exosomal RNA extraction methods

**Fig 3:**
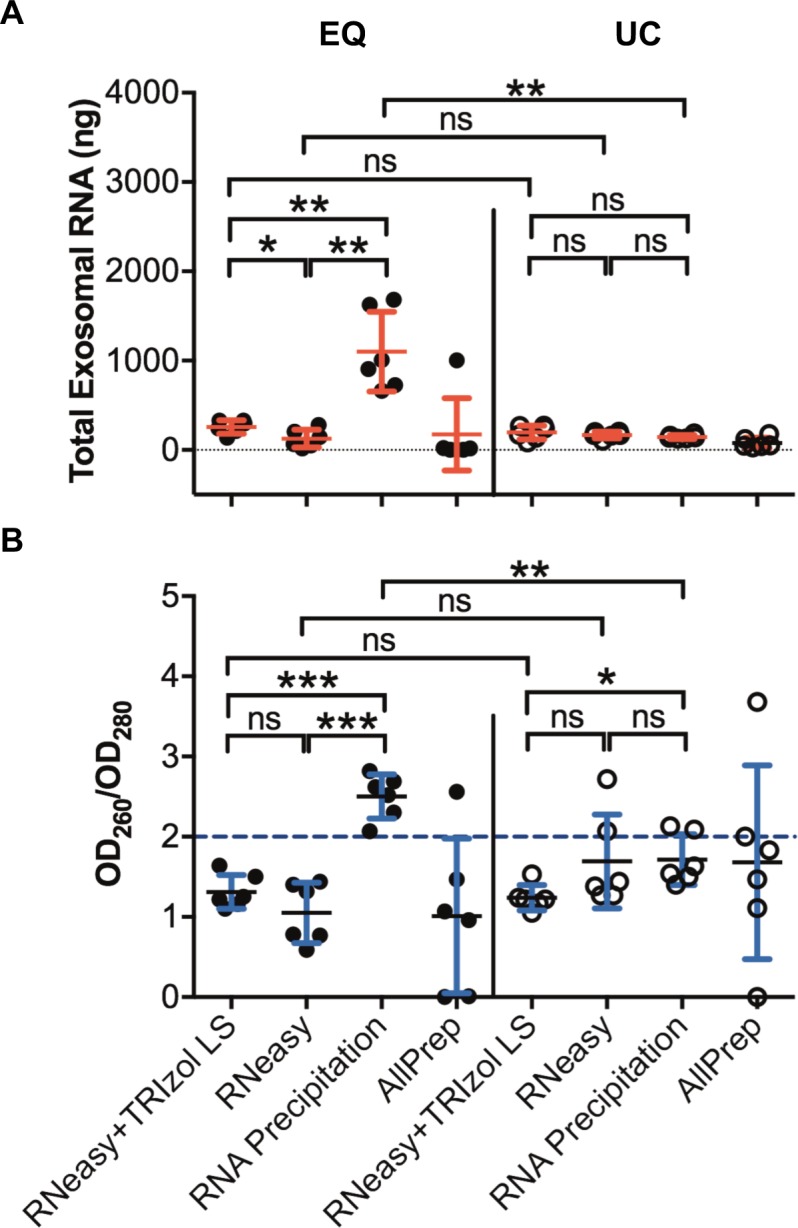
Comparison of sera exosomal RNA using four different RNA extraction methods. (A) Total RNA yield from ultracentrifugation (UC) and ExoQuick (EQ) treated samples using the RNeasy Mini Kit combined with TRIzol LS, the RNeasy Mini Kit alone, conventional RNA precipitation, and AllPrep DNA/RNA Mini Kit. (B) Demonstration of RNA quality measured by OD_260_/OD_280_ in EQ and UC treated samples. Data are shown as the mean ± SD from six independent patient samples. ***P<0.001, **P<0.01, *P<0.05, ns, not significant, student’s *t* test. Note: RNA could not be extracted from some samples using the AllPrep kit due to clogging.

For downstream applications (e.g. real time quantitative PCR (RT-qPCR), gene expression microarrays or RNA-Seq), both RNA quantity and quality are critical. RNA yield and OD_260_/OD_280_ ratios were therefore compared for exosomal RNA isolated using four different RNA extraction methods: the RNeasy Mini Kit combined with TRIzol LS, the RNeasy Mini Kit alone, conventional RNA precipitation, and the AllPrep DNA/RNA Mini Kit (Fig 3). Comparisons were performed for exosomes prepared from six healthy subjects using both the ultracentrifugation and the ExoQuick methods. RNA was successfully extracted in most cases except for 9 samples where RNA extraction was performed using the AllPrep DNA/RNA Mini Kit (Fig 3A and 3B), suggesting that this kit may not be suitable for exosomal RNA extraction. Average total RNA yields for ExoQuick-mediated enrichment of exosomes followed by RNA extraction with RNeasy with TRIzol LS, RNeasy alone, and RNA precipitation were: 256 ± 76, 129 ± 101, and 1100 ± 446 ng, respectively. Yields for specimens processed by ultracentrifugation then RNA extraction with RNeasy with TRIzol LS, RNeasy alone, and RNA precipitation were 195 ± 77, 165 ± 36, and 145 ± 22 ng, respectively (Fig 3A). Nanodrop analysis demonstrated that RNA yields were highest when ExoQuick was used in conjunction with standard RNA precipitation, but not with other methods (Fig 3A), each of which use RNeasy columns. These columns only permit extraction of RNA transcripts over 200 nt in length, whereas conventional precipitation methods retrieve a broader spectrum of RNA species including small RNAs. This is also supported by higher OD_260_/OD_280_ ratio of RNA extracted from ExoQuick samples using precipitation (Fig 3B). ExoQuick-treated samples generated significant more total RNA than samples processed using ultracentrifugation, although only when applied in combination with a traditional RNA precipitation method, suggesting that ExoQuick is more efficient in retrieving small RNAs than ultracentrifugation. The average quality and yield of RNA for other methods of RNA extraction was comparable for exosomes prepared by ExoQuick and ultracentrifugation (Fig 3A and 3B). In general, RNA yield and quality was most consistent and less variable when the RNeasy Mini Kit was combined with TRIzol LS, for both ExoQuick and ultracentrifugation methods.

### Determining the minimum serum volume for exosomal RNA isolation

**Fig 4:**
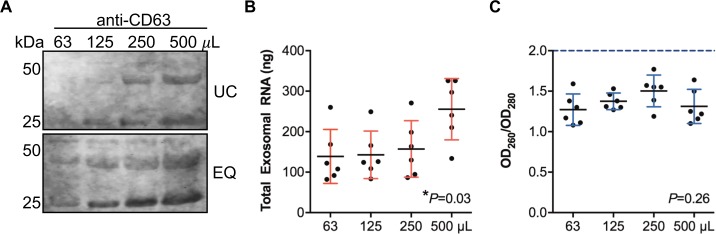
Minimal sera volume for exosome detection and RNA extraction. (A) Representative immunoblots for CD63 comparing exosome enrichment using ultracentrifugation (UC) and ExoQuick (EQ) among samples of varying volumes. (B) Scatter dot plots depict total exosomal RNA recovered per sample volume using EQ. (C) Scatter dot plots show RNA quality measured by OD_260_/OD_280_ per sample volume. Data are shown as the mean ± standard deviation (SD) from six independent patient samples. *P*-values were calculated using one-way ANOVA.

To determine the minimum volume of cell-free serum required for exosomal RNA isolation, 63 μL, 125 μL, 250 μL and 500 μL aliquots of cell-free serum were subjected to exosomal purification using both ultracentrifugation and ExoQuick (Fig 4A). Exosomal enrichment was confirmed using anti-CD63 Western blot analysis. CD63 signal was proportional to increasing sample volume, with a consistently stronger signal observed for ExoQuick treatment compared with ultracentrifugation, again indicating that ExoQuick is more efficient in enrichment of cell-free exosomes. Using ExoQuick, CD63 could be detected with as little as 63 μL starting serum, whereas 125 μL serum was required when using ultracentrifugation. RNA was isolated from each ExoQuick prepared sample using the RNeasy Mini Kit combined with TRIzol LS. The average yield of total RNA increased proportional to the sample volume (Fig 4B) and RNA quality was similar for all volumes (Fig 4C). While RNA yield was doubled when comparing RNA extracted from 500 μL with 250 μL serum, the relationship between sample volume and yield was non-linear at lower volumes, possibly due to that lower RNA concentrations reach the detection limit for the Nanodrop (~2 ng/μL).

### Effect of sample age on exosomal RNA quality and yield

**Fig 5:**
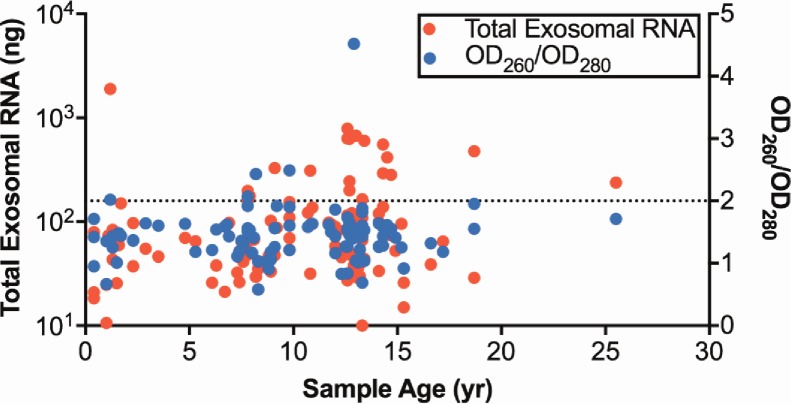
Exosomal RNAs are stable over two decades. Dot plots of total RNA yield and OD_260_/OD_280_ from 105 EQ-treated archival patient samples using the RNeasy Mini Kit combined with TRIzol LS. Storage time for each sample is indicated on the x-axis. The Spearman’s rank correlations for RNA yield *versus* storing time and OD_260_/OD_280_ *versus* storage time are 0.185 (*P* = 0.06) and 0.04 (*P* = 0.70), respectively.

We then tested the performance of our optimized exosomal RNA extraction on a cohort of clinical samples collected over the last 25 years. One hundred and five serum samples were processed using the ExoQuick reagent followed by RNA extraction using TRIzol LS combined with the RNeasy Mini Kit. The serum samples were stored at -80°C immediately after removing blood cells (see Materials and Methods), and had been stored for an average of 10.3 years (range: 0.3–25.5). Exosomal RNA was successfully extracted from all samples and crucially, neither RNA yield or RNA quality was correlated with sample age (Fig 5), indicating exosomal RNA is highly stable over extended periods of time when serum samples are stored at -80°C.

### Profiling exosomal transcriptomes using RNA-Seq

**Fig 6:**
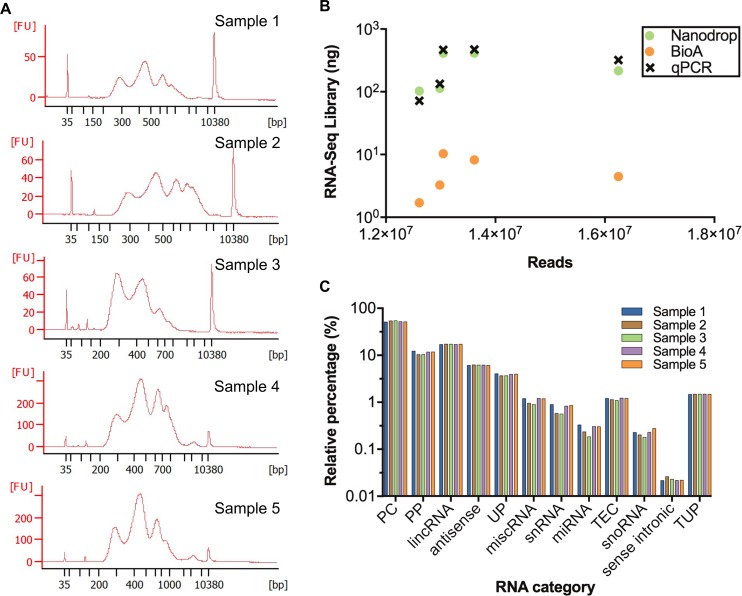
Exosomal RNA-Seq libraries from archival sera specimens. (A) Bioanalyzer results for 5 independent specimens demonstrate inserted size of exosomal RNAs. (B) Number of mapped reads generated from RNA-Seq libraries for 5 independent specimens with 20 ng exosomal RNAs. RNA-Seq library concentrations were calculated using three different methods: Nanodrop, BioAnalyzer (BioA), and RT-qPCR (qPCR). The Spearman’s rank correlations for Nanodrop *versus* reads, BioAnalyzer *versus* reads, and qPCR *versus* reads are 0.7 (*P* = 0.23), 0.6 (*P* = 0.35), and 0.7 (*P* = 0.23), respectively. (C) Relative abundance of biotypes detected for 5 exosomal RNA-Seq libraries from healthy female subjects. PC = protein coding, PP = processed pseudogene, lincRNA = long intergenic non-coding RNA, UP = unprocessed pseudogene, snRNA = small nuclear RNA, miRNA = microRNA, TEC = to be experimentally confirmed, snoRNA = small nucleolar RNA, TUP = transcribed unprocessed pseudogene.

To demonstrate that exosomal-derived RNA isolated using our optimized method can generate high quality RNA-Seq data, RNA-Seq libraries were constructed using 20 ng exosomal RNA from 5 unique sera specimens from healthy women. The average size of inserted exosomal RNAs was around 450 bp (Fig 6A). The concentrations of RNA-Seq libraries were measured by three different methods: Nanodrop, Bioanalyzer, and RT-qPCR. Total DNA amount measured by Nanodrop was more consistent with that measured by RT-qPCR for each sample, while the measurements from Bioanalyzer were much lower for all cases (Fig 6B). Seven nanograms of each RNA-Seq library was pooled and sequenced to generate 75 bp single-end reads. Despite using equal starting amounts of library based on Nanodrop measurements, the numbers of mapped raw reads were variable (Fig 6B). Correlation of library DNA amount to resulting mapped reads for Nanodrop or qPCR was higher than that for Bioanalyzer (r = 0.7 vs 0.6), suggesting that Nanodrop and qPCR are more suitable for measuring the concentration of RNA-Seq library than Bioanalyzer. RNA-Seq reads were mapped to the human genome and annotated using GENCODE25 data to classify exosomal transcripts into functional groups (Fig 6C). The most abundant biotype was protein-coding RNA (53%). Significant amount of long non-coding RNAs, including lincRNAs (17%) and antisense transcripts (6%), were also detected. Similar percentages of RNA with same biotypes were identified within those five samples (Fig 6C).

## Discussion

Although next-generation sequencing has transformed biomarker research, RNA-Seq profiling of liquid biopsy specimens is a relatively underexplored frontier. Our optimized protocol demonstrates that exosomes can be rapidly enriched from a very limited amount of fresh or archival human cell-free serum (under 63 μL) with particularly high efficiency when using a commercially available reagent–ExoQuick, followed by RNA extraction performed using TRIzol LS and the RNeasy Mini Kit. The exosomal RNA isolated using this protocol can be used for many downstream applications–RT-qPCR, gene expression microarray analysis, and, as demonstrated here, RNA-Seq analysis. RNA-Seq provides the most comprehensive characterization of exosomal transcriptomes, and can be used in functional modeling studies or in the discovery of biomarkers that could be used in the clinic for diagnosis, prognostication, or for assessing therapeutic response.

In contrast to RNA-Seq libraries built from RNA extracted from other sources (e.g. whole cells), the number of mapped reads in sera exosomal RNA-Seq libraries showed poorer correlations with quantifications performed using the Nanodrop, qPCR, and the Bioanalyzer. This could be because the exosomal RNA concentrations are typically lower, and near the lower limit of detection for Nanodrop. Nanodrop (or Qubit) measurements tend to overestimate nucleic acid concentrations and have greater variability while Bioanalyzer analyses typically underestimate concentration and demonstrate less variability. The accepted ‘gold-standard’ is an RT-qPCR measurement approached based on a standard curve of known DNA concentrations. Somewhat surprisingly, our results showed that for exosomal RNA-Seq libraries, Nanodrop quantification correlates higher with RT-qPCR measurements than Bioanalyzer. We therefore recommend estimating library concentration using Nanodrop and performing low-depth sequencing (e.g. using the Illumina MiniSeq) to accurately quantify the ratio of each library in the pool, and then adjusting the ratios as required before proceeding with deeper sequencing. This strategy will provide equal number of reads for each library within the pool, which is essential for quantitative analyses of exosomal RNAs across samples [42].

The ultracentrifugation protocol also performed well in our comparisons, but is only amenable to smaller studies, as substantially more serum samples can be processed in a drastically shorter time (at similar cost) using ExoQuick method (~18 h total processing time) compared with ultracentrifugation (~56 h total processing time). Additionally, use of the ExoQuick reagent retrieves more exosomes and exosomal RNAs than ultracentrifugation. Several methods of exosomal RNA extraction can be implemented, and it should be noted that precipitation of RNA by absolute ethanol recovered more RNA from ExoQuick-purified exosomes, but not ultracentrifugation-purified exosomes. Those exosomal RNAs had an average OD_260_/OD_280_ of 2.5, suggesting that small RNAs may be over-represented, as observed in previous studies [23, 24]. Therefore, ExoQuick treatment followed by traditional RNA precipitation is preferable if small RNAs are of interest. Crucially, age of the sample does not appear to influence the RNA yield, thus researchers can apply this technique to archival specimens that may have been stored for many years, although low temperature storage is likely to be critical (our specimens were stored at -80°C). Moreover, this optimized protocol can be readily adapted to the processing of other biospecimens such as urine, saliva, or stool and translation of this protocol into the clinical setting for laboratory testing may also be feasible.

In the past decade, exosomal components have been examined extensively to identify potential prognostic and diagnostic biomarkers for many human diseases, including cancer, but exosomal transcriptomes remain poorly characterized [29, 30]. As exosomes are stable over time, exosomal components uniquely secreted by diseased cells but not normal cells represent promising candidate biomarkers of human disease. Most exosomal RNAs are protein-coding and long non-coding RNAs, which are of particularly interest as novel biomarkers due to their higher cell-type specificity [38]. MiRNAs are also likely to be a key area of study as they represent a particularly promising group of candidate biomarkers due to their inherent stability. In summary, the current protocol provides an optimized strategy for novel biomarker discovery in liquid sera specimens by comprehensive exosomal RNA profiling that can be readily applied to a diverse array of human diseases.
